# Supplementary material for: Prognostic Value of C-Reactive Protein-to-Albumin Ratio in Head and Neck Cancer: A Meta-Analysis
Source: Diagnostics (Basel). 2021 Feb 26;11(3):403. doi: 10.3390/diagnostics11030403 (PMC7996835; doi:10.3390/diagnostics11030403)
Supplement: Supplementary file 1 [file diagnostics-11-00403-s001.zip › diagnostics-1099543-supplementary.pdf]

*Supplementary Materials*

# Prognostic Value of C-Reactive Protein-to-Albumin Ratio in Head and Neck Cancer: A Meta-Analysis

**Chih-Wei Luan, Hsin-Yi Yang, Yao-Te Tsai, Meng-Chiao Hsieh, Hsin-Hsu Chou and Kuo-Su Chen**

**Table S1.** The Newcastle–Ottawa Scale score of included studies.

| Study | Selection | | | | Comparability | | Outcome | | Non Response Rate | Score |
|---|---|---|---|---|---|---|---|---|---|---|
| | Is the Case Definition Adequate? | Representa tiveness of the Cases | Selection of Controls | Definition of Controls | Main Factor | Additional Factor | Ascertainment of Exposure | Same Method for Cases and Controls | | |
| Li et al. [1] | v | v | v | x | v | x | v | v | v | 7 |
| He et al. [2] | v | v | v | x | v | x | v | v | v | 7 |
| Zhang et al. [3] | v | v | v | x | v | v | v | v | v | 8 |
| Wang et al. [4] | v | v | v | x | v | v | v | v | v | 8 |
| Tao et al. [5] | v | v | v | x | v | x | v | v | v | 7 |
| Sun et al. [6] | v | v | v | x | v | v | v | v | v | 8 |
| Kuboki et al. [7] | v | v | x | v | v | v | v | v | v | 8 |
| Yu et al. [8] | v | v | x | x | v | v | v | v | v | 7 |
| Wang et al. [9] | v | v | v | x | v | v | v | v | v | 8 |
| Park et al. [10] | v | v | x | v | v | x | v | v | v | 7 |
| Fang et al. [11] | v | v | x | x | v | v | v | v | v | 7 |

**Table S2.** The detail for included studies.

| *p* Value | Clinical Staging with CAR | T Staging with CAR | N Staging with CAR | Independent Risk Factor | Analysis | Survival | CAR Unit | CAR Mean/Median | CAR Range | Study Period |
|---|---|---|---|---|---|---|---|---|---|---|
| Li et al. [1] | NA | NA | NA | Yes | Multivariate analysis | 5 years OS | CRP: mg/L albumin: g/L | NR | NR | Jan 2009 to Jan 2010 |
| He et al. [2] | <0.001 | <0.001 | <0.001 | Yes | Multivariate analysis | 5 years OS, DMFS | CRP: mg/L albumin: g/L | NR | NR | Mar 2000 to Oct 2013 |
| Zhang et al. [3] | NA | NA | NA | Yes | Multivariate analysis | 3 years OS | CRP: mg/L albumin: g/L | 0.093/0.036 | 0.002–4.594 | Nov 2009 to Feb 2012 |
| Wang et al. [4] | NA | <0.001 | =0.005 | Yes | Multivariate analysis | 5 years DMFS | CRP: mg/L albumin: g/L | NR/0.032 | NR | Dec 2009 to May 2011 |
| Tao et al. [5] | =0.024 | <0.001 | =0.842 | Yes | Multivariate analysis | 5 years OS | CRP: mg/L albumin: g/L | NR/0.138 | 0.010–1.932 | Jan 2007 to Dec 2012 |
| Sun et al. [6] | NA | NA | NA | Yes | Multivariate analysis | 5 years OS | CRP: mg/L albumin: g/L | NR/0.164 | NR | Jan 2008 to Oct 2011 |
| Kuboki et al. [7] | =0.008 | =0.1 | =0.1 | Yes | Multivariate analysis | 5 years OS, DFS | CRP: mg/L albumin: g/L | NR | NR | Feb 2003 to April 2012 |
| Yu et al. [8] | <0.05 | NA | <0.01 | Yes | Multivariate analysis | 5 years OS | CRP: mg/L albumin: g/L | NR/0.374 | 0.006–2.020 | May 2006 to Oct 2011 |
| Wang et al. [9] | <0.001 | <0.001 | <0.001 | Yes | Multivariate analysis | 5 years OS, DFS | CRP: mg/L albumin: g/L | NR | NR | Jan 2005 to Mar 2018 |
| Park et al. [10] | =0.002 | =0.002 | =0.006 | Yes | Multivariate analysis | 5 years OS, DFS | CRP: mg/L albumin: g/L | 0.137/NR | 0.005–1.124 | May 2004 to Dec 2011 |
| Fang et al. [11] | <0.001 | <0.001 | NA | Yes | Multivariate analysis | 5 years OS, DFS | CRP: mg/L albumin: g/L | NR/0.08 | 0.01–5.48 | Jan 2008 to Dec 2017 |

NA, not available; OS, overall survival; DFS, disease free survival; CAR, C-reactive protein to albumin ratio.

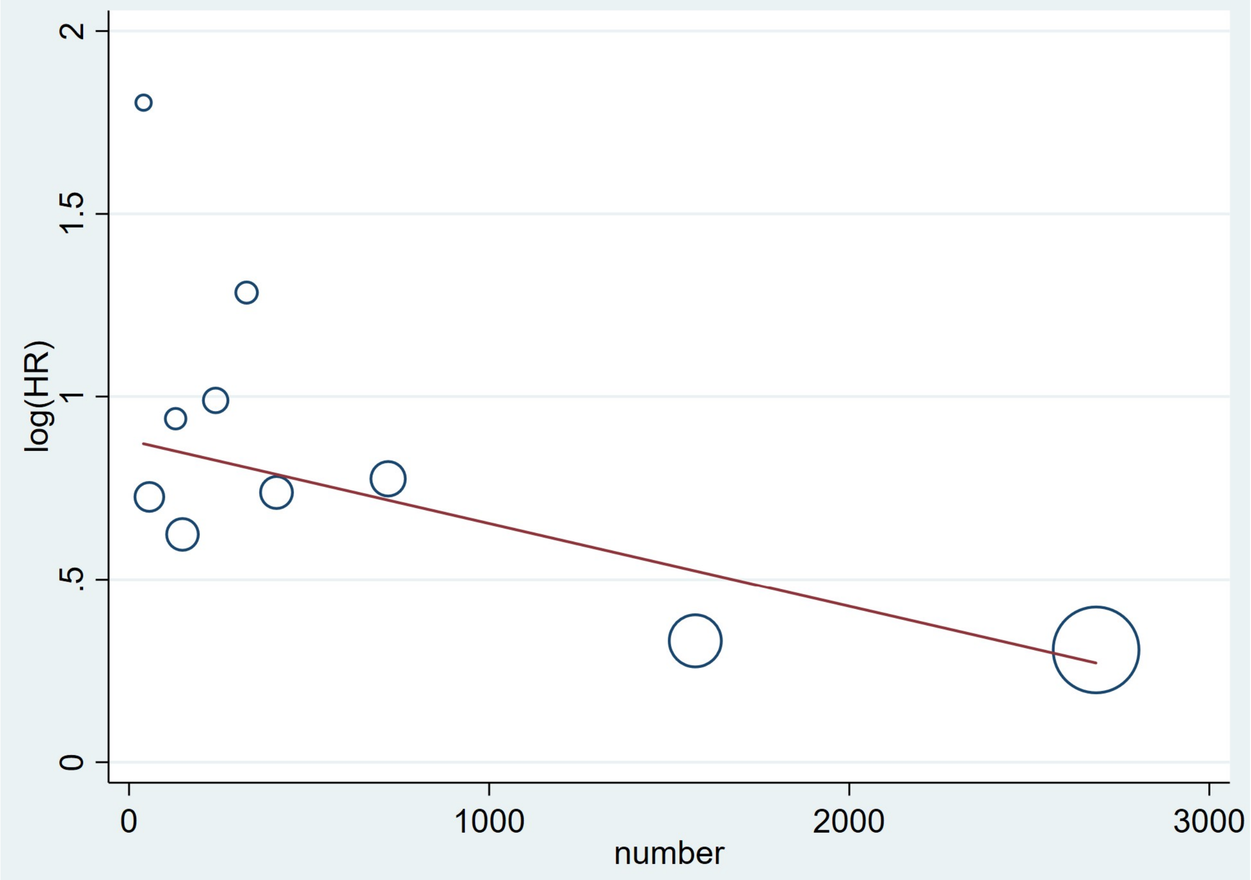


**Figure S1.** Meta-regression of sample size and overall survival. HR, hazard ratio.

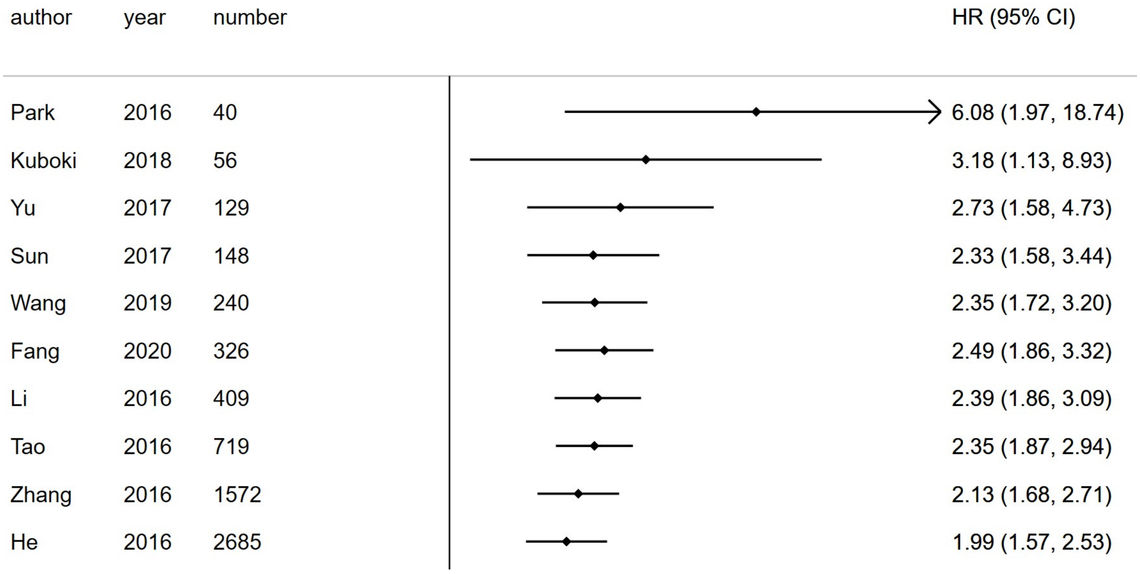


**Figure S2.** Cumulative meta-analysis by sample size.

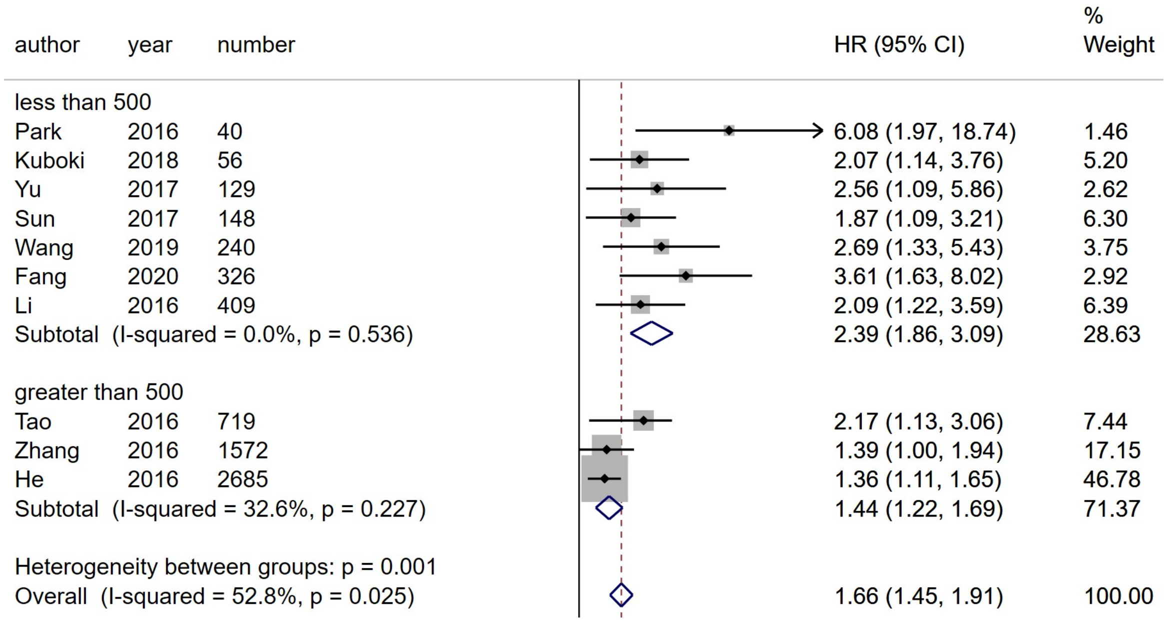


**Figure S3.** Forest plots for the subgroup analysis by sample size. OS, overall survival. CAR, C-reactive protein-to-albumin ratio. HNC, head and neck cancer.

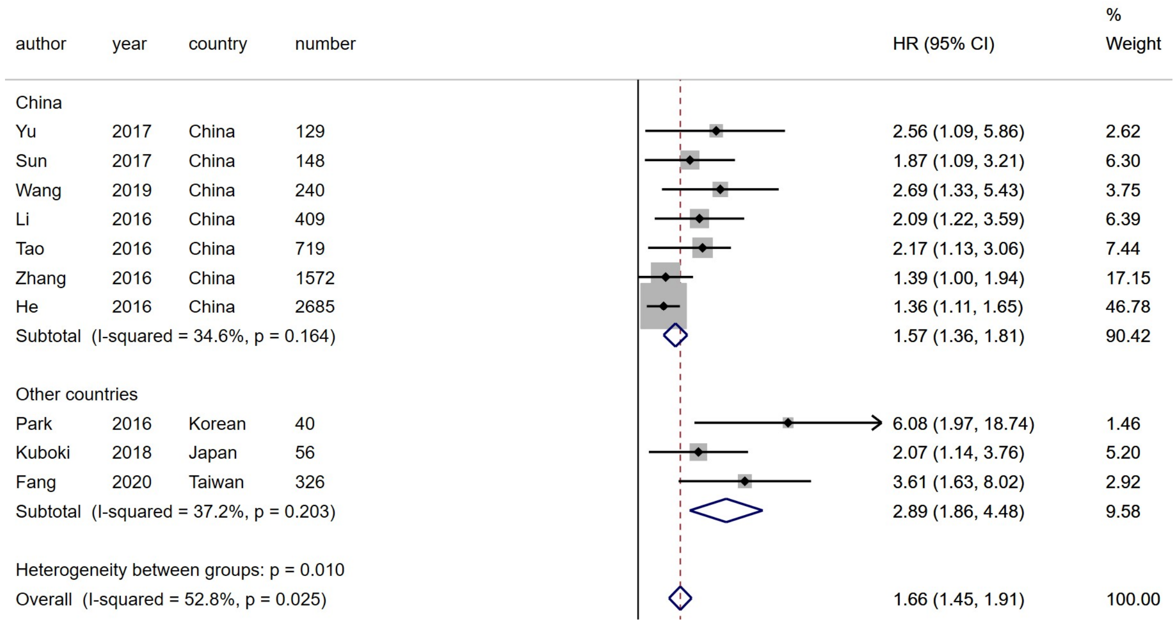


**Figure S4.** Forest plots for the subgroup analysis by country of origin.

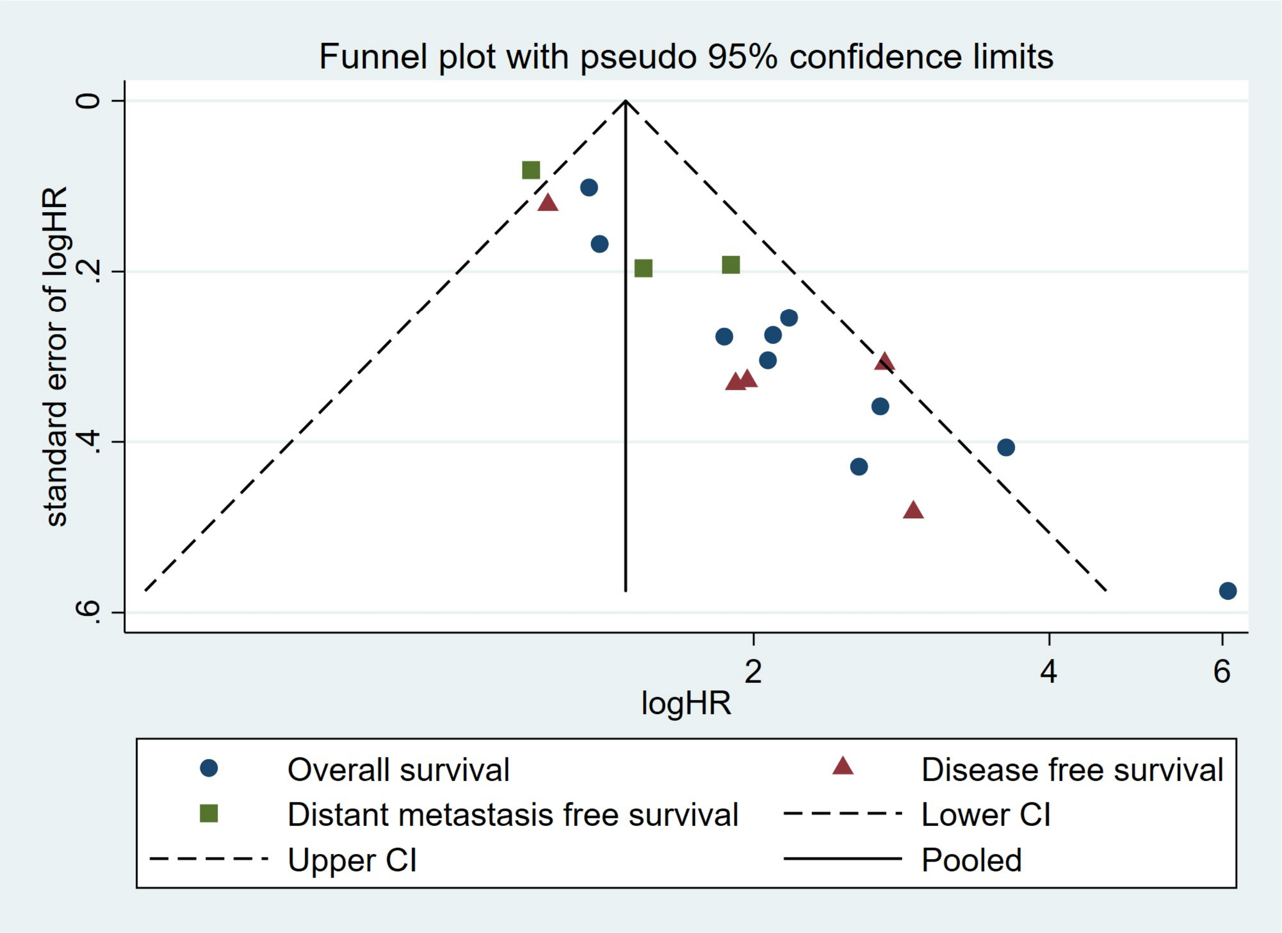


**Figure S5.** Funnel plots providing a graphic estimate of bias for all three outcomes. Each point represents a separate study. The two sloping lines represent the 95% confidence interval.

## Reference


1. Li, J.P.; Chen, S.L.; Liu, X.M; He, X.; Xing, S.; Liu, Y.-J.; Lin, Y.-H.; Liu, W.-L. A Novel Inflammation-Based Stage (I Stage) Predicts Overall Survival of Patients with Nasopharyngeal Carcinoma. *Int. J. Mol. Sci.* **2016**, *17*, 1900.
2. He, S.; Wang, Y.; Chen, H.; Yang, L.; Liang, S.; Lu, L.; Chen, Y. C-Reactive Protein/Albumin Ratio (CAR) as a Prognostic Factor in Patients with Non-Metastatic Nasopharyngeal Carcinoma. *J. Cancer* **2016**, *7*, 2360–2366.
3. Zhang, Y.; Zhou, G.Q.; Liu, X.; Chen, L.; Li, W.-F.; Tang, L.-L.; Liu, Q.; Sun, Y.; Ma, J. Exploration and Validation of C-Reactive Protein/Albumin Ratio as a Novel Inflammation-Based Prognostic Marker in Nasopharyngeal Carcinoma. *J. Cancer* **2016**, *7*, 1406–1412.
4. Wang, Y.; Yang, L.; Xia, L.; Chen, Y. High C-reactive protein/albumin ratio predicts unfavorable distant metastasis-free survival in nasopharyngeal carcinoma: A propensity score-matched analysis. *Cancer Manag. Res.* **2018**, *10*, 371–381.
5. Tao, C.J.; Chen, Y.Y.; Jiang, F.; Feng, X.-L.; Jin, Q.-F.; Jin, T.; Piao, Y.-F.; Chen, X.-Z. The C-reactive Protein/Albumin Ratio Is an independent Prognostic Factor for Overall Survival in Patients with Nasopharyngeal Carcinoma Receiving Intensity-Modulated Radiotherapy. *J. Cancer* **2016**, *7*, 2005–2011.
6. Sun, P.; Chen, C.; Xia, Y.; Bi, X.; Liu, P.; Zhang, F.; Yang, H.; An, X.; Jiang, W.; Wang, F. The Ratio of C-Reactive Protein/Albumin is a Novel Inflammatory Predictor of Overall Survival in Cisplatin-Based Treated Patients with Metastatic Nasopharyngeal Carcinoma. *Dis. Markers* **2017**, *2017*, 6570808.
7. Kuboki, A.; Kanaya, H.; Nakayama, T.; Konno, W.; Goto, K.; Nakajima, I.; Kashiwagi, T.; Hirabayashi, H.; Haruna, S.I. Prognostic value of C-reactive protein/albumin ratio for patients with hypopharyngeal and laryngeal cancer undergoing invasive surgery involving laryngectomy. *Head Neck* **2019**, *41*, 1342–1350.
8. Yu, S.T.; Zhou, Z.; Cai, Q.; Liang, F.; Han, P.; Chen, R.; Huang, X.-M. Prognostic value of the C-reactive protein/albumin ratio in patients with laryngeal squamous cell carcinoma. *OncoTargets Ther.* **2017**, *10*, 879–884.
9. Wang, Q.; Song, X.; Zhao, Y.; He, Q.; Shi, M.; Xu, P.; Ni, S.; Chen, Y.; Lin, J.; Zhang, L. Preoperative high c-reactive protein/albumin ratio is a poor prognostic factor of oral squamous cell carcinoma. *Future Oncol.* **2019**, *15*, 2277–2286.

10. Park, H.C.; Kim, M.Y.; Kim, C.H. C-reactive protein/albumin ratio as prognostic score in oral squamous cell carcinoma. *J. Korean Assoc. Oral Maxillofac. Surg.* **2016**, *42*, 243–250.
11. Fang, K.H.; Lai, C.H.; Hsu, C.M.; Huang, E.; Tsai, M.-S.; Chang, G.-H.; Lee, Y.-C.; Tsai, Y.-T. A retrospective study on the prognostic value of preoperative C-reactive protein to albumin ratio in patients with oral cavity squamous cell carcinoma. *PeerJ* **2020**, *8*, e9361.
